# METTL14 promotes tumorigenesis by regulating lncRNA OIP5-AS1/miR-98/ADAMTS8 signaling in papillary thyroid cancer

**DOI:** 10.1038/s41419-021-03891-6

**Published:** 2021-06-15

**Authors:** Xiaoping Zhang, Dan Li, Chengyou Jia, Haidong Cai, Zhongwei Lv, Bo Wu

**Affiliations:** 1 Department of Nuclear Medicine, Shanghai Tenth People’s Hospital, Tongji University, Shanghai, 200072 China; 2 Shanghai Center of Thyroid Diseases, Tongji University School of Medicine, Shanghai, 200072 China; 3 Zhuhai Precision Medical Center, Zhuhai People’s Hospital, Zhuhai Hospital Affiliated with Jinan University, Jinan University, Zhuhai, Guangdong 519000 China; 4 Clinical Nuclear Medicine Center, Tongji University School of Medicine, No. 301 Middle Yanchang Road, Shanghai, 200072 China; 5 Tongji University Medical College Imaging Clinical Medical Center, No. 301 Middle Yanchang Road, Shanghai, 200072 China; 6 Center of Thyroid, Department of General Surgery, Shanghai Jiao Tong University Affiliated Sixth People’s Hospital, Shanghai, 200233 China

**Keywords:** Tumour biomarkers, Oncogenes

## Abstract

**Background:**

Papillary thyroid cancer (PTC) is the most common type of cancer of the endocrine system. Long noncoding RNAs (lncRNAs) are emerging as a novel class of gene expression regulators associated with tumorigenesis. Through preexisting databases available for differentially expressed lncRNAs in PTC, we uncovered that lncRNA OIP5-AS1 was significantly upregulated in PTC tissues. However, the function and the underlying mechanism of OIP5-AS1 in PTC are poorly understood.

**Methods:**

Expression of lncRNA OIP5-AS1 and miR-98 in PTC tissue and cells were measured by quantitative real-time PCR (qRT-PCR). And expression of METTL14 and ADAMTS8 in PTC tissue and cells were measured by qRT-PCR and western blot. The biological functions of METTL14, OIP5-AS1, and ADAMTS8 were examined using MTT, colony formation, transwell, and wound healing assays in PTC cells. The relationship between METTL14 and OIP5-AS1 were evaluated using RNA immunoprecipitation (RIP) and RNA pull down assay. And the relationship between miR-98 and ADAMTS8 were examined by luciferase reporter assay. For in vivo experiments, a xenograft model was used to investigate the effects of OIP5-AS1 and ADAMTS8 in PTC.

**Results:**

Functional validation revealed that OIP5-AS1 overexpression promotes PTC cell proliferation, migration/invasion in vitro and in vivo, while OIP5-AS1 knockdown shows an opposite effect. Mechanistically, OIP5-AS1 acts as a target of miR-98, which activates ADAMTS8. OIP5-AS1 promotes PTC cell progression through miR-98/ADAMTS8 and EGFR, MEK/ERK pathways. Furthermore, RIP and RNA pull down assays identified OIP5-AS1 as the downstream target of METTL14. Overexpression of METTL14 suppresses PTC cell proliferation and migration/invasion through inhibiting OIP5-AS1 expression and regulating EGFR, MEK/ERK pathways.

**Conclusions:**

Collectively, our findings demonstrate that OIP5-AS1 is a METTL14-regulated lncRNA that plays an important role in PTC progression and offers new insights into the regulatory mechanisms underlying PTC development.

## Introduction

The incidence of thyroid cancer is constantly rising in the recent years, becoming the most common form of endocrine malignancy [1–3]. Papillary thyroid cancer (PTC) is the main thyroid cancer subtype that is most prevalent among young women and children. Detecting PTC at early stages results in a 5-year survival rate of 98.2% [4], however, this drastically drops to 59% when detected during late stages. Therefore, there is an increasing need for improved PTC diagnosis and treatment strategies.

N6-methyladenosine (m^6^A) methylation plays a vital role in maintaining the functions and characteristics of tumor cells during tumorigenesis and development. More importantly, many studies have reported that long non-coding RNAs (lncRNAs) harboring m^6^A modification have significant effects on target genes via protein-RNA interaction in a variety of tumors including cervical, breast, colorectal, and thyroid cancers [5, 6]. Notably, m^6^A is a dynamic modification, induced by methyltransferase-like 3 (METTL3) and methyltransferase-like 14 (METTL14) and removed by the RNA demethylases, fat-mass and obesity-associated protein (FTO), and alkylation repair homolog protein 5 (ALKBH5) [7]. Increasing evidences suggest that alterations in the expression of m^6^A methylation regulators, especially METTL14, can suppress cell proliferation and metastasis of cancers regulated by lncRNAs and mRNAs, such as XIST, SOX4, and PERP [8–10]. However, the mechanism underlying the role of METTL14 in PTC has not been elucidated.

LncRNAs have been well-characterized and are responsible for many tumor characteristics such as apoptosis and metastasis, and they collaborate with miRNAs to enable tumor invasion [11–13]. Additionally, lncRNAs contribute to cancer initiation, development, and progression, including PTC [14, 15], by modulating key tumor suppresser genes or oncogenes [16]. For example, lncRNA NEAT1 functions as an oncogene by sponging tumor-suppressive miRNAs in several types of cancerous cells. LncRNA SNHG15 acts as a competing endogenous RNA (ceRNA) to regulate YAP1-Hippo signaling pathway by sponging miR-200a-3p in PTC [4]. Hence, lncRNAs are indispensable for tumor progression and development. Recent studies have reported a marked upregulation in lncRNA OIP5-AS1 expression in many cancers [17–22], indicating the importance of lncRNA OIP5-AS1 in tumor development. However, its function and molecular mechanism of action in PTC has not been reported yet.

ADAMTS family of proteins is disintegrins and metalloproteinases with thrombospondin motifs playing a role in a variety of biological processes including extracellular matrix degradation, cell proliferation, apoptosis, migration/invasion, and angiogenesis. ADAMTS expression is dysregulated in a wide range of tumors. ADAMTS8, also referred to as METH-2, acts as an antiangiogenic factor in several tumors [23]. It has been identified as a novel tumor suppressor gene as high levels of ADAMTS8 is associated with poor prognosis among breast cancer patients [24] due to its role in promoting metastasis [25].

In the present study, through microarray analysis of PTC tissues, we identified lncRNA OIP5-AS1 as a target. We observed that OIP5-AS1 regulates ADAMTS8 via sponging miR-98. In addition, we also investigated the role of m^6^A Writer METTL14 in PTC and identified that METTL14 inhibits PTC proliferation and migration/invasion through OIP5-AS1/miR-98/ADAMTS8 axis and via MEK/ERK and EGFR signaling pathways. Hence, our study highlights the significant role of METTL14 and OIP5-AS1 in PTC tumor progression. METTL14 and OIP5-AS1 are identified to be important biomarkers and therapeutic targets for PTC.

## Materials and Methods

### Tissue collection

PTC tissue and adjacent thyroid tissues were collected from 72 patients from the Shanghai Tenth People’s Hospital affiliated with Shanghai Tongji University, and approved by the Research Ethics Committee of the Shanghai Tenth People’s Hospital (Shanghai, China). Patient studies were conducted in accordance with the Declaration of Helsinki. Patients did not receive local or systemic treatment prior to surgery. Written informed consent for research purposes was provided by all patients and their clinicopathological characteristics are summarized in Supplementary Table S1. Samples were frozen in liquid nitrogen immediately after surgical resection for mRNA and protein extraction. Kaplan–Meier curves were used to calculate overall survival rate of PTC patients. Patients were divided into high and low OIP5-AS1 or ADAMTS8 expression groups using the median expression level of OIP5-AS1 or ADAMTS8 as the cut-off point. Overall survival plot for high vs low expression of OIP5-AS1 and ADAMTS8 with a *p* value for Kaplan–Meier plot (log-rank test) and Cox proportional hazards model were used.

### Microarray analysis

Two pairs of PTC patient tissues were used for lncRNA integrated microarray analysis. The sample preparation and microarray hybridization were performed according to the manufacturer’s instruction. Briefly, mRNA was purified from total RNA after removal of rRNA (mRNA-ONLY Eukaryotic mRNA Isolation Kit, Epicentre, WI, USA), amplified and transcribed into fluorescent cRNA along the entire length of the transcript utilizing a random priming method. The arrays were scanned by Agilent Scanner (Agilent, CA, USA). Agilent Feature Extraction software (version 11.0.1.1) was used to analyze the acquired array images. LncRNA and mRNA expression patterns were revealed via hierarchical clustering. The threshold of significance was defined by *p* value.

### Cell culture

Human benign thyroid follicular epithelial cell line Nthy-ori 3-1 and PTC cell lines (TPC-1, K1, and BCPAP) were purchased from American Type Culture Collection (ATCC, Manassas, VA, USA) and BHP5-16 was purchased from ATCC (Rockville, MD, USA). All cell lines used in the study were cultured using Dulbecco’s modified Eagle’s medium (DMEM; GIBCO-BRL) containing 10% fetal bovine serum (FBS, Hyclone, Logan, UT, USA), in a humidified atmosphere with 5% CO_2_ at 37 °C.

### Plasmid construction and cell transfection

For OIP5-AS1 and ADAMTS8 overexpression, human full-length OIP5-AS1 and ADAMTS8 cDNAs were subcloned into the pcDNA3.1 (+) mammalian expression vector (Invitrogen) at the *Kpn*I and *Xho*I sites to generate pcDNA3.1-OIP5-AS1/ADAMTS8. All plasmids were purified using DNA Midiprep kit (Qiagen, Germany). K1 cells transfected with pcDNA3.1-OIP5-AS1 or ADAMTS8 were selected using G418 (geneticin) to generate stable clones. Cells transfected with the empty pcDNA3.1 (+) vector (pcDNA3.1-NC) was used as a negative control. siRNAs targeting OIP5-AS1, ADAMTS8, and METTL14 were synthesized by Genechem (Shanghai, China). For miR-98 overexpression and knockdown, miR-98 mimic, miR-98 inhibitor, or two corresponding scrambled miRNA negative controls (mimic-NC for miR-98 mimic and inhibitor-NC for miR-98 inhibitor) were also synthesized by Genechem. All transfections were performed using Lipofectamine 3000 reagent (Invitrogen) in HEK293T cells following the manufacturer’s instructions. Target sequences are listed in Supplementary Table S2.

### RNA extraction and quantitative real-time PCR (qRT-PCR)

Total RNA and miRNAs were extracted using TRIzol reagent (Invitrogen) and miRNeasy Mini Kit (Qiagen), respectively. For miRNA analysis, cDNA was obtained using TaqMan MicroRNA Reverse Transcription Kit (Applied Biosystems, Foster City, CA, USA). Expression of miR-98 was quantified by RT-PCR using TaqMan miRNA assay kit (Applied Biosystems). Data were normalized against U6 small nuclear RNA (U6 snRNA). For mRNA analysis, cDNA was synthesized using M-MLV reverse transcriptase (Invitrogen) and Oligo (dT) reverse transcription primers. Expression of OIP5-AS1 and ADAMTS8 was quantified by PCR using SYBR Green Real-Time PCR Master Mix (ThermoFisher, Waltham, MA, USA). Relative transcript abundance was determined by the 2^−ΔΔCt^ method and mRNA expression was normalized against GAPDH. All amplification assays were performed on a 7900HT Fast Real Time PCR machine (Applied Biosystems). Primer sequences used in PCR are listed in Supplementary Table S2.

### Western blot analysis

Total protein was extracted from cells and cerebral tissues using RIPA lysis buffer containing protease inhibitors. Samples (30 μg) were separated by 10% sodium dodecyl sulfate-polyacrylamide gel electrophoresis (SDS-PAGE) and subsequently transferred to a polyvinyl fluoride (PVDF) membrane (Millipore, Bedford, MA, USA). After blocking in 5% nonfat milk for 2 h, membranes were incubated overnight at 4 °C with primary antibodies recognizing Akt (Abcam, ab8805, 1:10000), p-Akt (Abcam, ab38449, 1:1000), MEK1/2 (Abcam, ab178876, 1:5000), ERK1/2 (Abcam, ab17942, 1:1000), phospho-MEK1/2 (Abcam, ab278723, Ser^217^/Ser^221^, 1:5000), phospho-ERK1/2 (Abcam, ab214362, Thr^202^/Tyr^204^, 1:10000), GAPDH (Abcam, ab8245, 1:5000), epidermal growth factor receptor (EGFR; BD Transduction Laboratories, 610016, 1:1000), phosphor-EGFR (Tyr^1086^; PA5-17281, Invitrogen, 1:1000), and METTL14 (Abcam, ab220030, 1:500). After washing in TRIS-buffered saline containing 0.1% Tween 20 (TBST), membranes were incubated with horseradish peroxidase-conjugated secondary antibodies (Santa Cruz Biotechnology, 1:10000) for 1 h at room temperature. Protein bands were visualized using chemiluminescence (ECL). The immunoblot signal was quantified with Image J software. Expression levels of target proteins in each sample were normalized against those of GAPDH.

### Luciferase reporter assay

Putative miR-98 binding sites in OIP5-AS1 and the 3′-untranslated region (UTR) of ADAMTS8 were predicted using StarBase [26] (http://starbase.sysu.edu.cn/mirMrna.php) and TargetScan [27] (http://www.targetscan.org), respectively. A wild-type (WT) and mutant (MUT) OIP5-AS1 sequence and a WT and MUT 3′-UTR fragment of ADAMTS8 containing the putative miR-98 binding site were synthesized by Genechem via site-directed mutagenesis using QuickChange Lightning kit (Stratagene, La Jolla, CA, USA). Mutations were confirmed by DNA sequencing. Constructs were cloned into the pmirGLO dual luciferase reporter vector (Promega, Madison, WI, USA) downstream of the luciferase reporter gene to generate OIP5-AS1-WT, OIP5-AS1-MUT, ADAMTS8-WT, and ADAMTS8-MUT luciferase reporter constructs. TPC-1 cells were seeded in 24-well plates and transfected with the luciferase reporter plasmids, miR-98 mimic, mimic-NC (nonspecific control for miR-98 mimic), miR-98 inhibitor, and inhibitor-NC (nonspecific control for miR-98 inhibitor), alone or in combination, for 48 h. Luciferase activity was determined using the Dual Luciferase Reporter Assay System (Promega). Relative luciferase activity was normalized against the Renilla luciferase internal control.

### Fluorescence in situ hybridization (FISH)

The specific fluorescence in FISH probe of OIP5-AS1 (5′-TGGCACTGCATGAGGGATTT-3′) and hsa-miR-98 probes (5′-CCACACACCAGGGAAAGTAGTAA-3′) were used to observe the co-localization of OIP5-AS1 and hsa-miR-98 in TPC-1 cells. TPC-1 cells were fixed with 4.0% paraformaldehyde and permeabilized with 0.2 M HCl supplemented with 40 μg/ml protease K. After 10 min incubation with 0.1 M triethanolamine and 0.25% aceticanhydride, the slides were pre-hybridized at 60 °C for 2 h, followed by hybridization overnight at 60 °C in a humidified chamber. Slides were then washed twice with 2 x SSC buffer, blocked with 20% sheep serum for 1 h, and incubated with anti-digoxigenin antibody (Abcam) for 1.5 h at room temperature. Finally, the slides were washed three times in TBST buffer and incubated in detection buffer in the dark for 10 min. Imaging was performed using an Olympus Fluoview laser scanning confocal microscope.

### RNA immunoprecipitation assay

TPC-1 cells (~1 × 10^7^) transfected with miR-98 mimic or mimic-NC for 48 h were washed with cold PBS and lysed with RNA immunoprecipitation (RIP) lysis buffer (EMD Millipore, Billerica, MA, USA). Cell lysates were incubated with magnetic beads conjugated with anti-Argonaute2 (AGO2) antibody (Millipore) or mouse IgG that served as a negative control (Millipore). Beads were collected and washed, and RNA was extracted in the presence of proteinase K. OIP5-AS1 was detected by RT-PCR and qRT-PCR. The cell lysate served as the input.

### Immunohistochemistry

Three-micrometer-thick continuous sections were deparaffinized using xylene I and II for 20 min, and dehydrated with an anhydrous ethanol gradient. Slides were soaked in 50 μl of 3% H_2_O_2_ for 20 min, then placed in 1 mM TRIS-EDTA (pH 9) water bath (100 °C) for 20 min and cooled to room temperature. After washing with PBS, 50 μl of primary antibody (#PA5-64274, rabbit anti-ADAMTS8/18/20, 1:100; Thermo Fisher Scientific) and anti-Ki67 (1:200, ab16667) was added and incubated at 4 °C overnight. An UltraView Universal DAB Detection Kit (#760-500, Ventana Medical Systems, Tucson, Arizona, USA) was used to detect rabbit primary antibodies used earlier, and slides were visualized using H_2_O_2_ substrate and DAB chromogen, producing a brown precipitate.

### Cell proliferation assays

Cell proliferation assays were performed using an MTT kit (Sigma, St. Louis, Mo) according to the manufacturer’s instructions. Briefly, cells were seeded in 6-well plates and maintained in media containing 10% FBS for 2 weeks. Colonies were fixed with methanol and stained with 0.1% Crystal Violet (Sigma, St. Louis, Mo). Visible colonies were manually counted.

### Transwell assays

Transfected cells were harvested for invasion and migration assays. For migration assays, a 24-well Boyden chamber (8.0 μm pore size; Corning, Corning, NY, USA) coated with fibronectin (Roche Custom Biotech, Indianapolis, IN, USA) was used. 48 h post transfection, TPC-1 or K1 cells (6 × 10^4^) was resuspended in 100 μl of serum-free DMEM and seeded into the upper chamber of a transwell, while the lower chamber was filled with 600 μl of DMEM and 10% FBS. After incubation at 37 °C with 5% CO_2_ for 24 h, the upper chamber was cleaned with a cotton swab to remove cells on the upper surface of the membrane, and migrated cells remaining at the bottom of the membrane were fixed with 4% paraformaldehyde and stained with 1% Crystal Violet. For cell invasion assays, a Matrigel-precoated transwell chamber (Corning) was used, and subsequent steps were performed as described above. Images were obtained using a DMI4000B microscope (Leica, Wetzlar, Germany) and the number of cells was counted in five random fields (200×).

### Wound healing assay

Wound healing assays were performed to assess cell migration using the Ibidi 2-well culture insert system (Ibidi, Martinsried, Germany) according to the manufacturer’s instructions. Briefly, after the insert was placed into a 12-well plate, TPC-1 or K1 cells (6 × 10^4^/ml) were seeded in each well. At 50–60% confluency, cells were transfected with exogenous miRNAs or plasmids and cultured at 37 °C and 5% CO_2_ until 90–95% confluent. The insert was gently removed using sterile tweezers, and the well was filled with serum-free DMEM. Cultures were then incubated for 24 h and photographed using an Axio Observer Z1 phase contrast microscope (Zeiss, Oberkochen, Germany).

### Cellular fractionation

Cellular fractionation was performed to determine the subcellular localization of OIP5-AS1. Briefly, K1 and TPC-1 cells were lysed with RSB buffer (10 mM Tris-HCl, pH 7.4, 2.5 mM MgCl_2_, 100 mM NaCl) containing 4 mg/ml digitonin (BN2006, Thermo Fisher Scientific). After centrifugation, the supernatant was collected as the cytosolic extract. The remaining nuclear pellet was washed five times with RSB buffer and then lysed with RIPA buffer. GAPDH and U6 served as markers for the cytosolic and nuclear fraction, respectively.

### RNA pull-down assay

RNA pull-down assay was performed with RNA-Protein Pull-Down Kit (Pierce, USA) according to the manufacturer’s instructions. Briefly, full-length of OIP5-AS1 was transcribed in vitro using Large Scale RNA Production Systems (Promega, USA) and labeled with Biotin using Biotin RNA Labeling Mix (Roche, Switzerland). Then 1 mg cell lysates extracted from K1 cells was incubated with 3 μg purified biotinylated transcripts for 1 h at 4 °C with rotation. Streptavidin agarose beads were added to the protein lysate to precipitate the RNA–protein complex. The beads were washed three times and boiled in sodium dodecyl sulfate (SDS) buffer to retrieve proteins for western blot analysis.

### Xenograft model

BALB/c nude mice were purchased from the Chinese Science Academy (Shanghai, China). All animal experiments were made in the animal laboratory in Shanghai Tenth People’s Hospital and performed according to the guidelines with the approval of the Institutional Animal Care and Use Ethics Committee of Shanghai Tenth People’s Hospital affiliated with Shanghai Tongji University, China. In brief, mice were anesthetized with an intraperitoneal (i.p) injection of pentobarbital sodium (25 mg/kg) and placed on a surgical thermostator. Mice were randomly allocated. Then OIP5-AS1/pcDNA3.1-NC/OIP5-AS1 + si-ADAMTS8 was stably transfected into K1 cells, si-OIP5-AS1/si-NC/si-OIP5-AS1 + ADAMTS8 was stably transfected into TPC-1 cells. Transfected cells were then injected into 4-week-old female BALB/c nude mice (*n* = 6/per group) at a density of 1 × 10^6^ cells, and the mice were maintained under SPF conditions. Tumor size and volume were checked and measured regularly. Thirty days after implantation, mice were euthanized by cervical dislocation and tumors were harvested for further analysis.

### Statistical analysis

Data were presented as means ± standard error of the mean (SEM) from at least three independent experiments. Statistical analysis was performed using GraphPad Prism 5.01 software (GraphPad Software, La Jolla, CA, USA). Results from two different groups were compared by Student’s *t*-test. Significant differences between three or more groups were analysed using one-way or two-way ANOVA followed by the Bonferroni post hoc test. Differences with a *p* value less than 0.05 were deemed statistically significant.

## Results

### OIP5-AS1 and ADAMTS8 are upregulated in PTC tissues

**Fig. 1:**
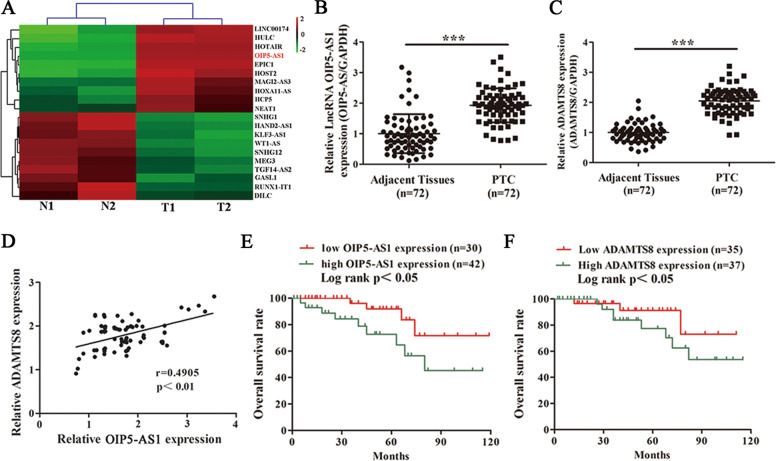
OIP5-AS1 and ADAMTS8 are upregulated in PTC. **A** Heatmap illustrating the 20 differentially regulated lncRNAs between two pairs of tumor tissues and corresponding adjacent tissuesfrom PTC patients (fold change >2, *p* < 0.05). The mark in red denotes lncRNA OIP5-AS1. **B**, **C** Representative OIP5-AS1 and ADAMTS8 expression in tumor sections of 72 patients with PTC were detected by qRT-PCR. **D** Pearson correction of OIP5-AS1 and ADAMTS8 were analysed (*n* = 72). **E**, **F** Survival rate of PTC patients was analyzed by Kaplan–Meier method. Log-rank tests were used to determine statistical significance. ***p < 0.001.

To identify lncRNAs involved in PTC, we analyzed total RNA from two pairs of PTC tissues with a noncoding RNA microarray (Arraystar). In total, we identified 20 lncRNAs altered in their expression, of which ten were upregulated and ten were downregulated in PTC tissues. OIP5-AS1 was the most upregulated lncRNA and hence, was selected for further functional validation (Fig. 1A). We assessed the expression of OIP5-AS1 and ADAMTS8 in 72 pairs of PTC tissues and adjacent normal tissues. We observed a significant increase in the expression of OIP5-AS1 and ADAMTS8 in PTC tissues compared to adjacent normal tissues (Fig. 1B, C). Besides, OIP5-AS1 positively correlated with ADAMTS8 by linear-regression analysis (Fig. 1D). Robust staining for ADAMTS8 was observed, indicating high expression, in PTC tissues compared with adjacent normal tissues (Fig. S1A). To verify the prognostic value of OIP5-AS1 and ADAMTS8 in PTC patients, Kaplan–Meier method was carried out. There was a steady decline in patient survival with higher levels of OIP5-AS1 and ADAMTS8 expression. The 5-year survival rate for patients with high OIP5-AS1 and ADAMTS8 expression was lower than that of patients with low OIP5-AS1 and ADAMTS8 expression (Fig. 1E, F). Next, the correlation between OIP5-AS1 or ADAMTS8 expression and clinical features of PTC patients was analyzed. We observed that higher expression of OIP5-AS1 and ADAMTS8 correlated with tumor size, lymph node metastasis, and TNM stage (Supplementary Table S1).

### OIP5-AS1 and ADAMTS8 regulate PTC cell proliferation, migration/invasion ability, and activate downstream EGFR and MEK/ERK pathways

**Fig. 2:**
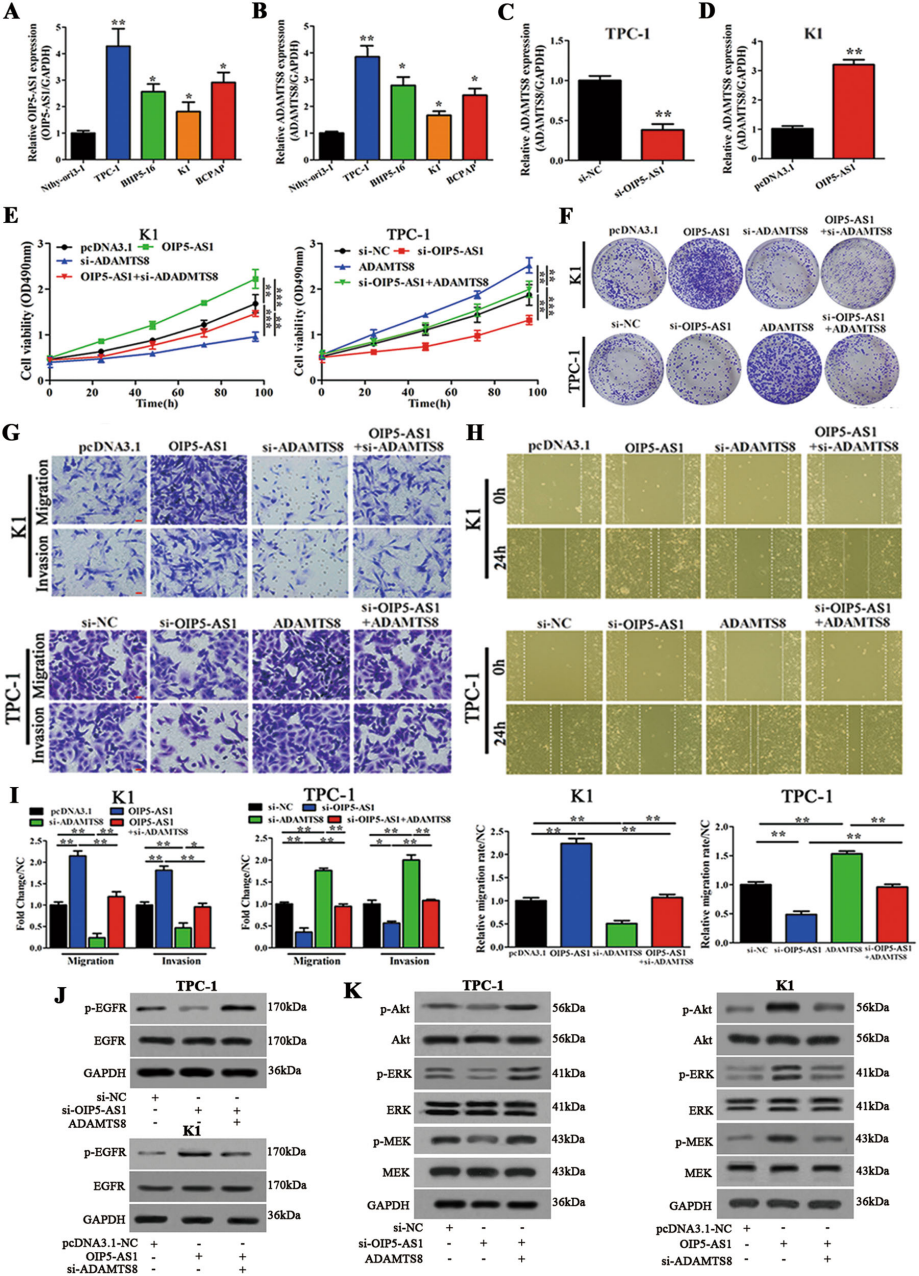
OIP5-AS1 and ADAMTS8 regulate PTC cell proliferation, migration/invasion and activate EGFR and MEK/ERK pathways in vitro. **A**, **B** OIP5-AS1 and ADAMTS8 expression in four PTC cell lines (TPC-1, BHP5-16, K1, and BCPAP), and in a normal human thyroid epithelial cell line Nthy-ori3-1 were analyzed by qRT-PCR (*n* = 3). **C**, **D** qRT-PCR analysis of ADAMTS8 expression levels in K1 cells and TPC-1 cells, K1 cells were transfected with si-NC/si-OIP5-AS1, while TPC-1 cells were transfected with pcDNA-3.1/OIP5-AS1 (*n* = 3). **E–I** K1 cells were co-transfected with OIP5-AS1 and si-ADAMTS8, while TPC-1 cells were co-transfected with si-OIP5-AS1/ADAMTS8 (*n* = 3). **E**, **F** MTT and colony-forming growth assays were performed to determine the proliferation of K1 and TPC-1 cells harboring the different vectors indicated (*n* = 3). **G** Transwell assays were performed to determine the migration and invasion capacity of K1 and TPC-1 cells (*n* = 3). Scale bar = 50 μm. **H** Wound healing assays were performed to assess the migratory capacity of K1 and TPC-1 cells (*n* = 3). **I** Migration and invasion abilities (fold change of migrated or invaded) were calculated, compared with the different vectors in K1 and TPC-1cells. The migratory activity (wound healing) was calculated and compared with those at 0 h (*n* = 3). **J** Western blot analysis of phosphorylated EGFR and total EGFR in TPC-1 and K1 cells (*n* = 3). **K** Phosphorylated and total Akt, ERK, and MEK levels measured by western blot analysis in TPC-1 and K1 cells (*n* = 3).*p < 0.05, **p < 0.01, and ***p < 0.001.

To determine the underlying function of OIP5-AS1 and ADAMTS8 in PTC cells, functional assays were designed and carried out. OIP5-AS1 lncRNA expression and ADAMTS8 mRNA expression were evaluated in four different PTC cell lines (TPC-1, BHP5-16, K1, and BCAP) and in a human benign thyroid follicular epithelial cell line (Nthy-ori3-1). We observed that OIP5-AS1 and ADAMTS8 expression were significantly higher in PTC cell lines when compared to Nthy-ori3-1 cell line (Fig. 2A, B). Among PTC cell lines, TPC-1 presented the highest OIP5-AS1 and ADAMTS8 expression while K1 presented the lowest. Hence, we used TPC-1 cells for knockdown and K1 cells for overexpression of both factors in our subsequent experiments. The knockdown efficiency of OIP5-AS1 and ADANTS8 in TPC-1 and overexpression efficiency in K1 cells were assessed by qRT-PCR (Fig. S1B, C). Si-OIP5-AS1-#1 and si-ADAMTS8-#2 showed higher knockdown efficiencies and hence, were chosen for subsequent experiments (termed as si-OIP5-AS1 and si-ADAMTS8 from now on). Next, we determined the ADAMTS8 expression in OIP5-AS1 knockdown TPC-1 cells and OIP5-AS1 overexpressing K1 cells by qRT-PCR. Our results showed that OIP5-AS1 overexpression could promote ADAMTS8 expression in TPC-1 cells. On the contrary, OIP5-AS1 knockdown has the opposite effects in K1 cells (Fig. 2C, D).

To determine the functional consequence of OIP5-AS1 and ADAMTS8 in PTC cell proliferation and migration/invasion, we performed MTT assay, colony formation assay, transwell, and wound healing assays (Fig. 2E–I). OIP5-AS1 overexpression promotes cell proliferation and migration/invasion in K1 cells, and OIP5-AS1 knockdown has the opposite effect in TPC-1 cells. ADAMTS8 overexpression or knockdown showed similar results in PTC cell proliferation and migration/invasion. More importantly, the cell proliferation and migratory/invasive capacity upon OIP5-AS1-overexpression was abrogated by ADAMTS8 knockdown in K1 cells. On the contrary, the cell proliferation and migratory/invasive capacity of OIP5-AS1 knockdown was restored by ADAMTS8 overexpression in TPC-1 cells. Together, these results indicate that OIP5-AS1 and ADAMTS8 participate in the proliferative, migratory, and invasive capabilities of PTC cells.

To identify the pathway underlying the effect of OIP5-AS1 and ADAMTS8, we evaluated the levels of phosphorylated EGFR, total EGFR, p-Akt, total Akt, p-ERK, ERK, p-MEK, and MEK by western blot in PTC cell lines (Fig. 2J, K). Expression of these proteins was decreased upon OIP5-AS1 knockdown in TPC-1 cells, and the overexpression of ADAMTS8 restored this inhibition. In contrast, expression of these markers was increased in OIP5-AS1 overexpressing K1 cells, and ADAMTS8 knockdown suppressed this effect. These results suggest that PTC progression via OIP5-AS1 and ADAMTS8 is mediated by activation of both EGFR and MEK/ERK pathways.

### OIP5-AS1 promotes PTC cell proliferation and tumor growth in vivo

**Fig. 3:**
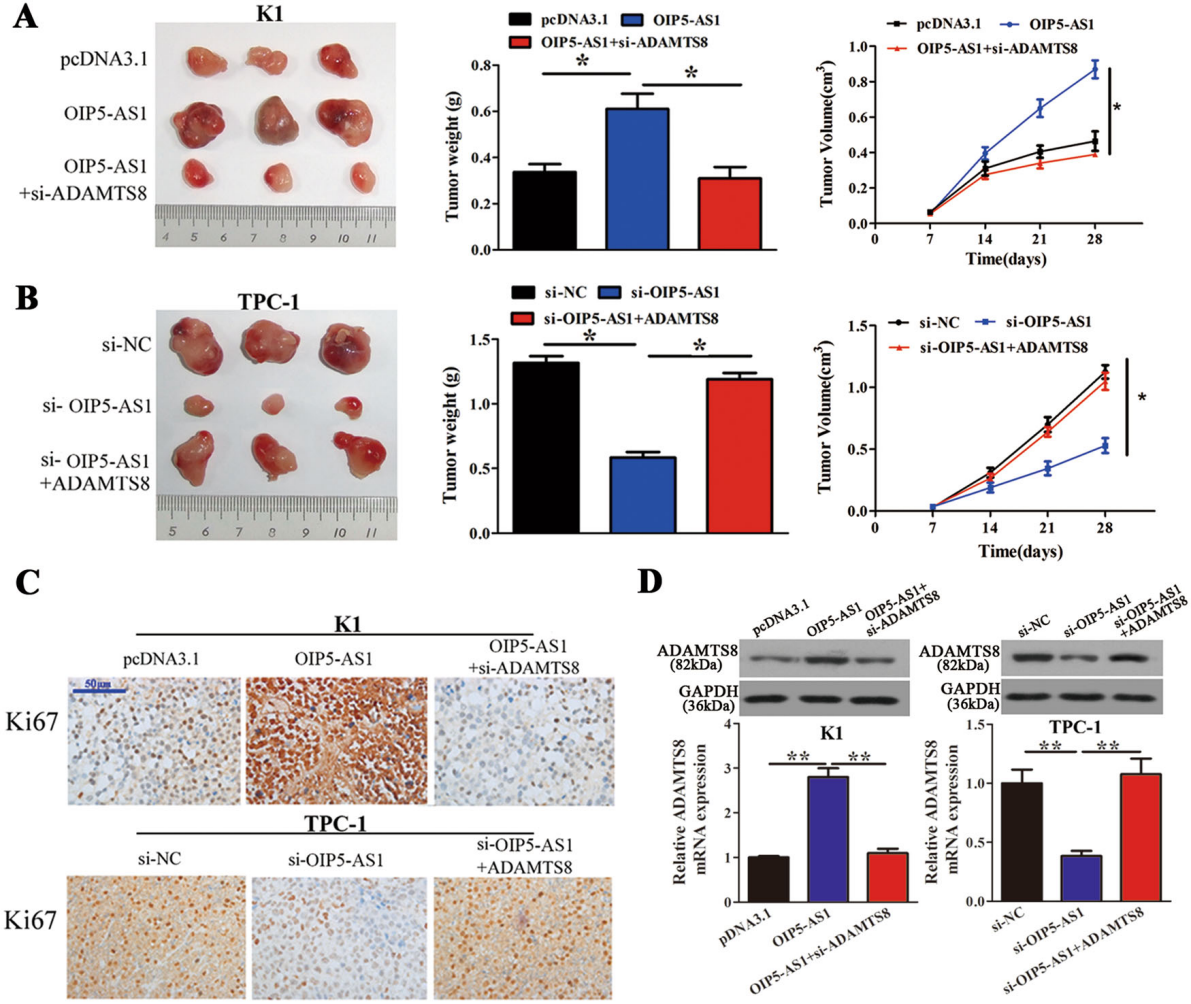
OIP5-AS1 regulates PTC cell growth in vivo. **A** Tumor weight and growth curves (*n* = 6) were measured after injection of K1 cells transfected with OIP5-AS1 and si-ADAMTS8. **B** Tumor weight and growth curves (*n* = 6) were measured after injection of TPC-1 cells transfected with si-OIP5-AS1 and ADAMTS8. **C** Ki67 staining to assess the proliferation capacity of K1 cells and TPC-1 cells (*n* = 6). Scale bar = 50 μm. **D** Western blot and qRT-PCR analyses of ADAMTS8 expression levels in K1 and TPC-1 cells (*n* = 6). *p < 0.05 and **p < 0.01.

To evaluate whether OIP5-AS1 serves as a PTC-related lncRNA in vivo, 1 × 10^6^ transfected cells were injected into 6-week-old BALB/c nude mice, and tumor weight and volume were assessed following injection. In mice that received OIP5-AS1-overexpressing K1 cells (Fig. 3A), tumor weight and volume were significantly increased when compared to the negative control, and this increase was lost in tumors from mice that received ADAMTS8 knockdown cells. In addition, mice that received OIP5-AS1 knockdown TPC-1 cells (Fig. 3B) showed significantly lower tumor weight and volume, this effect was restored upon ADAMTS8 overexpression. IHC staining using Ki67 antibodies implied that OIP5-AS1 overexpression could promote the proliferation ability of tumors, and this induction was lost upon ADAMTS8 knockdown. Similarly, OIP5-AS1 knockdown decreases the proliferation ability of tumors, and this reduction was lost upon ADAMTS8 overexpression (Fig. 3C). Lastly, ADAMTS8 protein and mRNA expression were evaluated in K1 and TPC-1 cells (Fig. 3D). ADAMTS8 expression was significantly increased when OIP5-AS1 was overexpressed, and this effect was lost upon ADMATS8 knockdown in K1 cells; whereas ADAMTS8 expression was reduced upon OIP5-AS1 knockdown, and this effect was lost upon ADAMTS8 overexpression in TPC-1 cells. These results indicate that expression levels of ADAMTS8 and OIP5-AS1 are directly related and reduction in ADAMTS8 levels reduces the effect of OIP5-AS1 in PTC cell proliferation.

### OIP5-AS1 functions through the ceRNA sponging pattern of miR-98

**Fig. 4:**
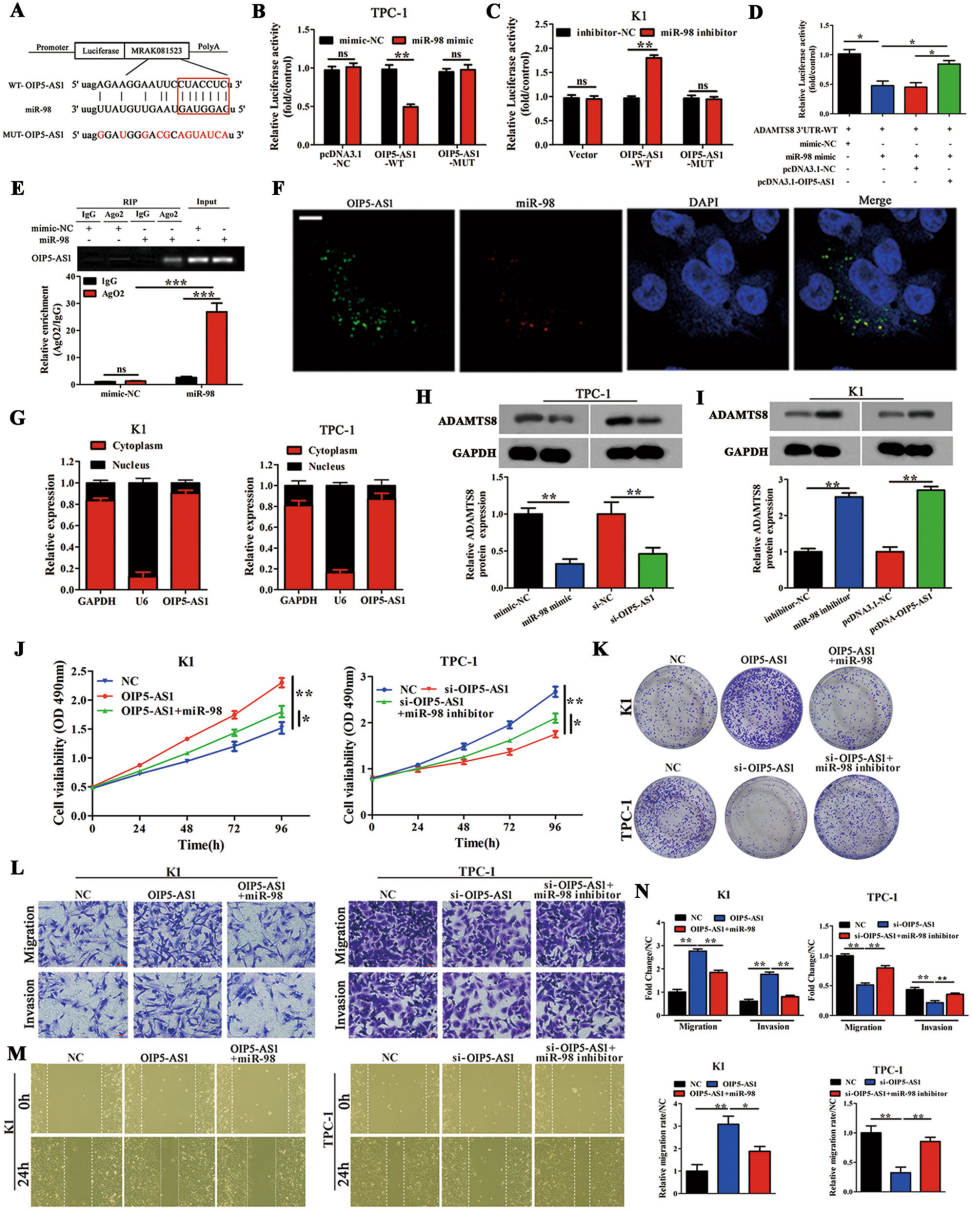
OIP5-AS1 is a target of miR-98 and regulates its expression. **A** Wild-type (OIP5-AS1-WT) and mutant (OIP5-AS1-MUT) OIP5-AS1 with mutations at the predicted miR-98 binding site (*n* = 3). **B**, **C** A luciferase reporter vector carrying OIP5-AS1-WT or OIP5-AS1-MUT (or the empty vector) was co-transfected into TPC-1 cells with miR-98 mimic or mimic-NC (**B**) or miR-98 inhibitor or inhibitor-NC (**C**) as indicated. Relative luciferase activity was measured at 48 h after transfection (*n* = 3). **D** The luciferase reporter vector carrying ADAMTS8 3′-UTR-WT was co-transfected into TPC-1 cells with miR-98 mimic, mimic-NC, pcDNA3.1-OIP5-AS1, and pcDNA3.1-NC, alone or in combination as indicated. Relative luciferase activity was measured at 48 h after transfection (*n* = 3). **E** TPC-1 cells were transfected with miR-98 mimic or mimic-NC for 48 h, and association between OIP5-AS1 and miR-98 was assessed by RNA immunoprecipitation assay (*n* = 3). **F** Fluorescence in situ hybridization assay with cells stained for OIP5-AS1 and miR-98 (*n* = 3) (Scale bar = 20 μm). **G** Relative lncRNA OIP5-AS1 expression level in the cytoplasm and nucleus of the K1 cells was determined by qRT-PCR assay (*n* = 3). **H**, **I** Western blot analysis of ADAMTS8 protein levels following treatment of TPC-1 cells with miR-98 mimic or si-OIP5-AS1, and K1 cells with miR-98 inhibitor or pcDNA3.1-OIP5-AS1. GAPDH was used as an internal control (*n* = 3). **J**, **K** MTT and colony-forming growth assays were performed to determine the proliferation of K1 and TPC-1 cells (*n* = 3). **L** Transwell assays were performed to determine the migration and invasion capacity of K1 and TPC-1 cells (*n* = 3). Scale bars = 50 μm. **M** Wound healing assays were performed to assess the migratory capacity of K1 and TPC-1 cells (*n* = 3). **N** The migration and invasion abilities and the migratory activity (wound healing) were calculated (*n* = 3). *p < 0.05, **p < 0.01, ***p < 0.001, ns not significance.

To further understand the various binding targets of OIP5-AS1; we conducted immunoprecipitation studies in TPC-1 and K1 cells overexpressing OIP5-AS1 using a probe specific to OIP5-AS1 and a control probe (Fig. S1D). The putative candidate miRNAs binding to OIP5-AS1 were predicted using StarBase (http://starbase.sysu.edu.cn/). The enrichment of OIP5-AS1 and miRNAs was detected by qRT-PCR and normalized to the corresponding levels in the control probe. Many miRNAs were enriched in the presence of OIP5-AS1 (Fig. S1D). Among these, miR-98 was the most highly enriched, and hence, we used this miRNA as a potential candidate for further studies. Mutations were introduced at the miR-98 binding site as shown in Fig. 4A. Luciferase reporter activity was evaluated in TPC-1 and K1 cells 48 h post co-transfection with OIP5-AS1-WT, OIP5-AS1-MUT, or empty vector along with either miR-98 mimic/mimic-NC or miR-98 inhibitor/inhibitor-NC (Fig. 4B–D). The results indicated that relative luciferase activity was significantly reduced in the presence of miR-98 but doubled in the presence of OIP5-AS1. Given the significance of OIP5-AS1-miR-98 in PTC cells, we further analyzed the clinical relevance of OIP5-AS1-miR-98 and found that the expression of OIP5-AS1 negatively correlates with miR-98 expression in PTC patients (Fig. S1E).

We next evaluated the interaction between OIP5-AS1 and miR-98 using RIP assays. Cells transfected with miR-98 mimic were enriched for both AGO2 and the negative control IgG compared with mimic-NC (Fig. 4E). Since the subcellular localization of OIP5-AS1 may play an important role in understanding the mechanisms underlying OIP5-AS1 function, we performed RNA FISH and nuclear-cytoplasmic fractionation assays. These results revealed that there is a co-localization between OIP5-AS1 and miR-98 in the cytoplasm of TPC-1 and K1 cells (Fig. 4F, G). Furthermore, ADAMTS8 expression was evaluated in K1 and TPC-1 cells, and the results indicated that ADAMTS8 protein expression was significantly decreased in the presence of miR-98 mimic and OIP5-AS1 knockdown TPC-1 cells. Conversely, ADAMTS8 expression was increased in the presence of miR-98 inhibitor and OIP5-AS1-overexpressing K1 cells, demonstrating that OIP5-AS1 is a target of miR-98 (Fig. 4H, I).

To determine the effect of OIP5-AS1 and miR-98 on cell proliferation and migration/invasion in PTC cells, we performed MTT assay, colony formation assay, transwell, and wound healing assays. Cell proliferation and migration/invasion of OIP5-AS1 expressing cells were reduced upon addition of miR-98 mimic in K1 cells, and inhibition of miR-98 significantly improved these effects in TPC-1 cells (Fig. 4J–N). Moreover, miR-98 mimic could abolish the promotion of OIP5-AS1 overexpression on cell proliferation and migration/invasion. In contrast, miR-98 inhibitor could attenuate the reduction of OIP5-AS1 knockdown on cell proliferation and migration/invasion. These results indicate that miR-98 acts as a negative regulator of OIP5-AS1 function and OIP5-AS1 promotes the cell proliferation and migration/invasion of PTC cells by targeting miR-98.

### MiR-98 regulates ADAMTS8 expression and cell proliferation, migration/invasion by directly targeting its 3′-UTR region

**Fig. 5:**
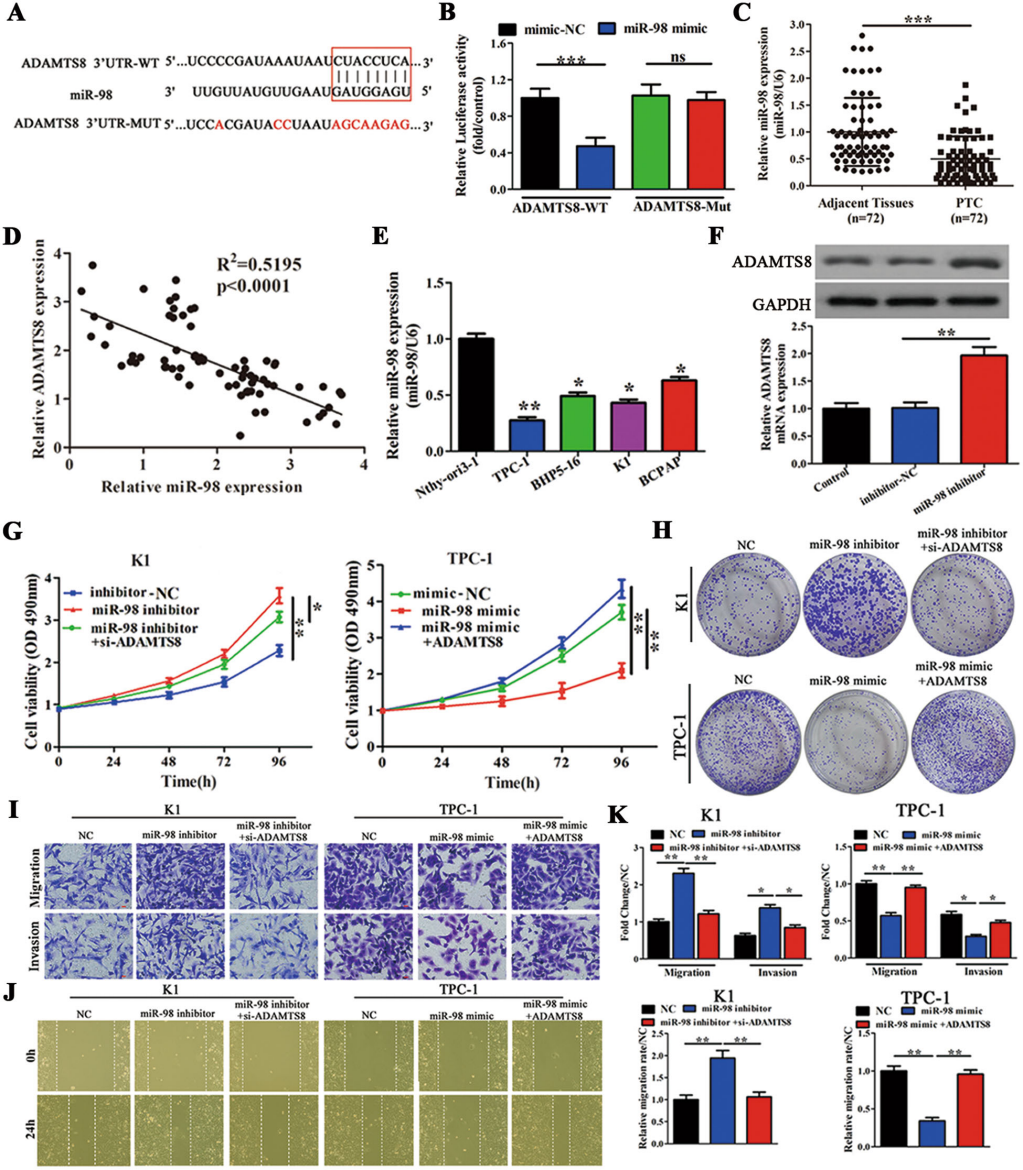
ADAMTS8 is a direct target of miR-98 and is involved in miR-98-mediated cell proliferation and migration/invasion. **A** WT 3′-UTR of ADAMTS8 (ADAMTS8 3′-UTR-WT) and a mutant 3′-UTR of ADAMTS8 with mutations at the predicted miR-98 binding site (ADAMTS8 3′-UTR-MUT) (*n* = 3). **B** A luciferase reporter vector carrying ADAMTS8 3′-UTR-WT or ADAMTS8 3′-UTR-MUT (or the empty vector) was co-transfected with miR-98 mimic or mimic-NC, and relative luciferase activity was measured 48 h post transfection (*n* = 3). **C** qRT-PCR analysis of miR-98 expression in 72 paired PTC tissues and corresponding adjacent tissues. **D** Pearson correction of miR-98 and ADAMTS8 were analysed (*n* = 72). **E** qRT-PCR analysis of miR-98 expression in four PTC cell lines (TPC-1, BHP5-16, K1, and BCPAP), and in Nthy-ori3-1 cells (*n* = 3). **F** Western blot and qRT-PCR analyses of ADAMTS8 expression levels in K1 cells transfected with inhibitor-NC or miR-98 inhibitor (*n* = 3). **G**, **H** MTT and colony-forming growth assays were performed to determine the proliferation of K1 and TPC-1 cells (*n* = 3). **I** Transwell assays were performed to determine the migration and invasion capacity of K1 and TPC-1 cells (*n* = 3). Scale bars = 50 μm. **J** Wound healing assays were performed to assess the migratory capacity of K1 and TPC-1 cells (*n* = 3). **K** The migration and invasion abilities and the migratory activity (wound healing) were calculated and compared to the different vectors (*n* = 3). *p < 0.05, **p < 0.01, and ***p < 0.001.

Mutations were introduced into the miR-98 binding site of ADAMTS8 (Fig. 5A). When WT and mutated ADAMTS8 cells were co-transfected with empty or miR-98 mimic, a significant reduction in luciferase activity was observed upon addition of miR-98 mimic in WT cells, but there was no difference in mutated ADAMTS8 cells (Fig. 5B). Next, miR-98 expression was evaluated in 72 patient samples. RT-PCR results indicate that miR-98 expression was significantly lower in PTC tissues than in normal adjacent tissues (Fig. 5C). We then analyzed the clinical relevance of miR-98 and ADAMTS8 in PTC patients and observed that the expression of ADAMTS8 was negatively associated with the expression of miR-98 (Fig. 5D). MiR-98 expression was significantly lower in PTC cell lines when compared to the normal Nthy-ori3-1 cell line. Of note, TPC-1 cell lines displayed the most significant differences in miR-98 expression (Fig. 5E). However, ADAMTS8 expression was increased when miR-98 was inhibited in K1 cells (Fig. 5F). These results conclusively prove that ADAMTS8 is a direct target of miR-98.

Next, we used immunofluorescence to assess ADAMTS8 expression in K1 and TPC-1 cells (Fig. S2). These results indicated that miR-98 inhibitor promoted ADAMTS8 expression, and the addition of si-ADAMTS8 reduced these levels in K1 cells. Furthermore, miR-98 mimic reduced ADAMTS8 expression and overexpression of ADAMTS8 reversed this effect in TPC-1 cells. MTT assay, colony formation assay, transwell, and wound healing assay were performed to detect the cell proliferation and migratory and invasive capacity of miR-98 in PTC cells. In K1 cells, miR-98 inhibitor significantly increased cell proliferation and migration/invasion, and ADAMTS8 knockdown reduced these effects. In TPC-1 cells, miR-98 mimic significantly reduced cell proliferation and migration/invasion. However, overexpression of ADAMTS8 reversed these effects (Fig. 5G–K). These results indicate that addition of ADAMTS8 rescues the miR-98 mediated inhibition of cell proliferation, migration, and invasion in PTC cells.

### METTL14 interacts with LncRNA OIP5-AS1 and regulates its expression

**Fig. 6:**
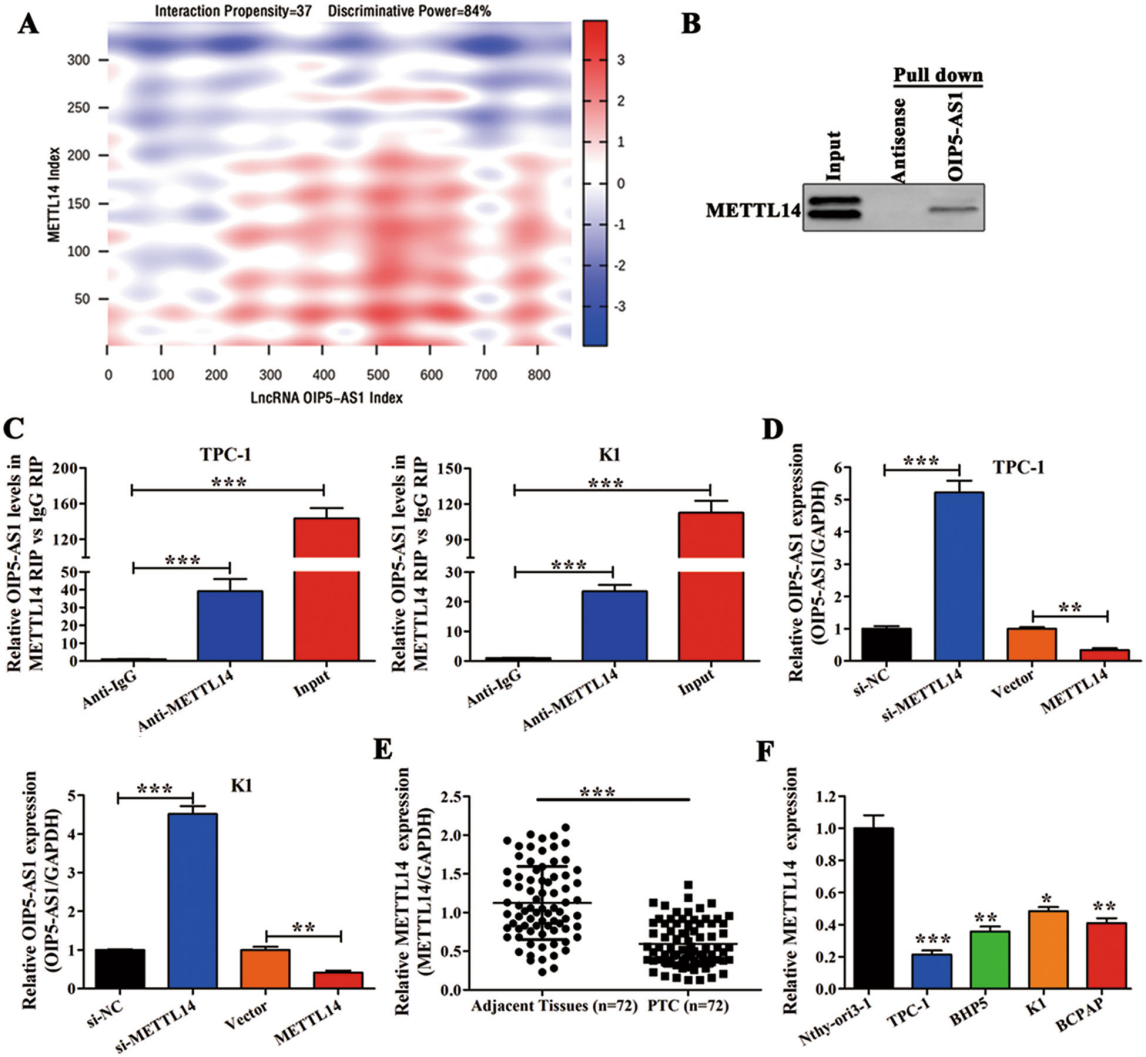
METTL14 interacts with lncRNA OIP5-AS1 and regulates its expression. **A** CatRAPID prediction result of lncRNA OIP5-AS1 and METTL14 shown as a heat-map. The X- and Y-axes represent the indexes of the RNA and protein sequences, respectively. The colors of the heat-map indicate the interaction score of the individual amino acid and nucleotide pairs. The total sum represents the overall interaction score. Interaction parameters between OIP5-AS1 and METTL14: interaction propensity = 37 and discriminative power = 84%. **B** Western blot analyses following RNA pull-down assays in TPC-1 cells confirmed the interaction between OIP5-AS1 and METTL14. Input: total proteins. Pull down: proteins immunoprecipitated by RNA (*n* = 3). **C** RIP assay followed by qRT-PCR in TPC-1 and K1 cells (*n* = 3). **D** qRT-PCR analysis of lncRNA OIP5-AS1 in TPC and K1 cells, which were transfected with si-NC/si-METTL14 and Vector/METTL14 (*n* = 3). **E** qRT-PCR analysis of miR-98 expression in 72 paired human PTC tissues and corresponding adjacent tissues. **F** qRT-PCR analysis of miR-98 expression in four PTC cell lines (TPC-1, BHP5-16, K1, and BCPAP), and in Nthy-ori3-1 (*n* = 3). *p < 0.05, **p < 0.01, and ***p < 0.001.

After establishing the role of OIP5-AS1 in PTC, we assessed the possibility of RNA-binding proteins that could interact with OIP5-AS1 to mediate its effects in PTC cells. We used catRAPID (http://service.tartaglialab.com/page/catrapid_group) [28] to identify putative RNA-binding proteins specific to OIP5-AS1. We discovered an interaction between OIP5-AS1 and METTL14 with an interaction propensity of 37 and discriminative power of 84%. The prediction result between OIP5-AS1 and METTL14 is shown as a heat-map in Fig. 6A. To verify this interaction, we performed RNA pull-down assays followed by western blot in TPC-1 cells (Fig. 6B). As expected, RIP assays showed an enrichment of OIP5-AS1 in precipitates from METTL14 fraction when compared to a fraction with control IgG (negative control) in TPC-1 and K1 cells (Fig. 6C). Next, we detected the OIP5-AS1 and METTL14 expression by qRT-PCR in TPC-1 and K1 cells. The results indicated that METTL14 overexpression or knockdown could significantly downregulate or upregulate OIP5-AS1 expression, respectively, but OIP5-AS1 overexpression or knockdown did not affect METTL14 expression in TPC-1 and K1 cells (Fig. 6D and Fig. S1F). Subsequently, we assessed METTL14 expression in PTC tissues and cell lines and observed that METTL14 expression was lower in both PTC tissues and cell lines (Fig. 6E, F). We also found that METTL14 expression was the highest in K1 cells and lowest in TPC-1 cells. Overall, these results demonstrate that METTL14 associates with OIP5-AS1 to regulate its expression in PTC and the expression of METTL14 is lower in PTC tissues and cell lines.

### METTL14 promotes PTC cell proliferation and migration/invasion and activates EGFR, Akt, and MEK/ERK pathways downstream through OIP5-AS1

**Fig. 7:**
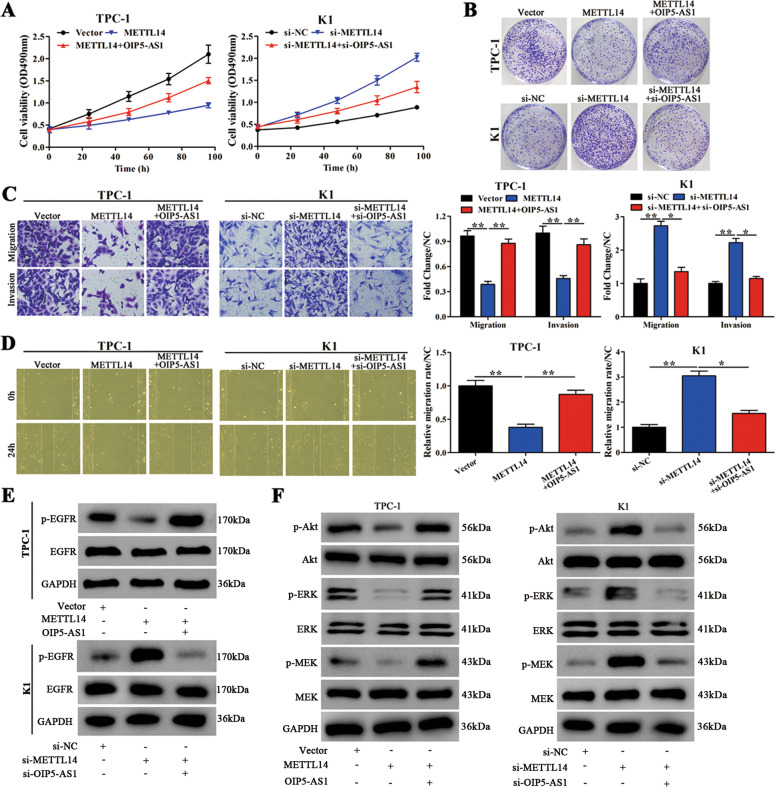
METTL14 promotes PTC cell proliferation, migration/invasion, and activate downstream EGFR, Akt, and MEK/ERK pathways through lncRNA OIP5-AS1. **A**, **B** MTT and colony-forming growth assays were performed to determine the proliferation ability of K1 and TPC-1 cells (*n* = 3). **C** Transwell assays were performed to determine the migration and invasion capacity of K1 and TPC-1 cells. Scale bars = 50 μm. The migration and invasion abilities (fold change of migrated or invaded) were calculated and compared to the different vectors in K1 and TPC-1 cells (*n* = 3). **D** Wound healing assays were performed to assess the migratory capacity of K1 and TPC-1 cells. The migratory activity (wound healing) was calculated and compared to vectors at 0 h (*n* = 3). **E** Western blot analysis of phosphorylated EGFR and total EGFR in TPC-1 and K1 cells (*n* = 3). **F** Phosphorylated and total Akt, ERK, and MEK levels measured by western blot analysis in TPC-1 and K1 cells (*n* = 3). *p < 0.05 and **p < 0.01.

To determine if the suppression of lncRNA OIP5-AS1 by METTL14 could influence PTC cell proliferation and migration/invasion, MTT assay, colony formation assay, transwell, and wound healing assays were performed (Fig. 7A–D). In TPC-1 cells, METTL14 overexpression significantly decreased cell proliferation and migration/invasion, and OIP5-AS1 overexpression alleviated this inhibition. In K1 cells, METTL14 knockdown significantly increased cell proliferation and migration/invasion. However, OIP5-AS1 knockdown significantly reduced this effect. Together, these results indicate that METTL14 expression rescues OIP5-AS1-mediated induction of cell proliferation, migration, and invasion in PTC cells.

Subsequently, we assessed whether METTL14 could affect EGFR, Akt, and MEK/ERK pathways by western blot analysis (Fig. 7E, F). In TPC-1 cells, METTL14 overexpression decreased p-EGFR, p-Akt, p-ERK, and p-MEK protein levels, and this reduction was alleviated upon OIP5-AS1 overexpression. In K1 cells, METTL14 knockdown induced expression of p-EGFR, p-Akt, p-ERK, and p-MEK and this increase was lost upon OIP5-AS1 knockdown. These results suggest that PTC progression via OIP5-AS1 and METTL14 is mediated by activation of EGFR, Akt, and MEK/ERK signaling pathways.

## Discussion

There has been mounting evidences certifying lncRNAs as important oncogenes or tumor suppressors playing a role in regulating the expression of tumor-related genes and the functions of tumor-related pathways in cancer [29, 30]. LncRNAs are overexpressed in a variety of cancers, including lncRNA DGCR5 [31] and DANCR [32] in lung cancer, lncRNA H19 in melanoma [33], and lncRNA TUG1 in ovarian cancer [34]. With respect to PTC, lncRNAs BANCR, HOTTIP, Gas5, and HOTAIR have all been implicated in disease prognosis [35–38]. Our study aimed to investigate the potential involvement of lncRNA OIP5-AS1 in mechanisms associated with PTC progression.

We observed a significant increase in OIP5-AS1 expression in PTC tissues compared with adjacent normal tissues and speculated that the upregulation of OIP5-AS1 may promote an aggressive tumor phenotype. Our data confirmed that OIP5-AS1 promotes PTC progression by enhancing PTC cell proliferation, migration/invasion as well as tumor growth in vivo. These results indicate that OIP5-AS1 may participate in a potential pathway that functions in PTC progression. We also simultaneously identified ADAMTS8 to be significantly upregulated in PTC. Given that ADAMS genes are overexpressed, mutated, or epigenetically silenced in various tumors, we sought to specifically evaluate expression of ADAMTS8 in our samples. Similar to OIP5-AS1, ADAMTS8 was also significantly elevated in PTC tissues. Furthermore, patients with higher levels of OIP5-AS1 and ADAMTS8 exhibited lower life expectancy over a 5-year period. These results suggest that both OIP5-AS1 and ADAMTS8 perform important functional roles in PTC progression. To confirm this hypothesis, loss-of-function and gain-of-function experiments were conducted for both OIP5-AS1 and ADAMTS8 in PTC cell lines. We found that cell proliferation and migration/invasion properties were significantly reduced upon OIP5-AS1 or ADAMTS8 knockdown, while overexpression of these factors significantly enhanced these properties. We next sought to mechanistically identify the pathways involved in OIP5-AS1- and ADAMTS8-mediated PTC progression. Choi et al. showed that ADAMTS8 acts as a tumor suppressor by antagonizing EGFR-MEK-ERK signaling [39]. Furthermore, EGFR, Akt pathways are activated in anaplastic and follicular thyroid cancers [40]. In our present study, p-EGFR, p-Akt, p-ERK, and p-MEK protein expression were significantly higher in samples containing OIP5-AS1, while silencing of OIP5-AS1 resulted in significantly lower levels of these proteins. These results suggest that OIP5-AS1 and ADAMTS8 support PTC progression via activation of EGFR, Akt, and MEK/ERK pathways.

Previous studies have shown that lncRNAs can reverse their target miRNA functions in human malignancies, and miRNAs, a subtype of noncoding RNAs, have also been implicated in malignant tumors [4]. Herein, miR-98 binding sites in OIP5-AS1 and ADAMTS8 genes were predicted using StarBase and Target Scan, respectively. We found that OIP5-AS1 was significantly downregulated in the presence of miR-98 and upregulated when miR-98 was inhibited. Furthermore, miR-98 inhibition significantly enhanced viability and the invasive/migratory potential of OIP5-AS1. Overexpression of ADAMTS8 significantly enhanced cell migration/invasion in the presence of miR-98 mimics, thereby offsetting its negative effects. These results imply that ADAMTS8 is responsible for miR-98-mediated proliferation, migration, and invasion. Our in vitro results were also supported by in vivo subcutaneous studies performed on a BALB/C nude mouse model. Specifically, knockdown of OIP5-AS1 caused a decrease in tumor tissue volume, and relative ADAMTS8 levels were significantly reduced upon OIP5-AS1 knockdown.

M^6^A has emerged as a popular modification in variety of cancers, which is proved to play crucial roles in regulating cell growth, invasion, and metastasis via controlling RNA splicing, translation, and stability [8]. Notably, recent studies discovered that m^6^A modification was also present in ncRNAs [10]. METTL14, acting as the central component of m^6^A complex, has been verified to be dysregulated and involved in the initiation and progression of various malignancies [9]. In this study, we observed a decrease of METTL14 in PTC tissues and cell lines. Through catRAPID and RNA pull-down assays, we found that METTL14 could bind to OIP5-AS1 and regulate its expression. Moreover, we demonstrated that METTL14 overexpression could suppress PTC cell proliferation, migration/invasion, and OIP5-AS1 overexpression alleviated METTL14-mediated effects on PTC tumor progression. On the contrary, METTL14 knockdown promotes PTC cell proliferation, migration/invasion, and OIP5-AS1 knockdown attenuated the effects of METTL14. These results indicate that METTL14 suppresses PTC cell proliferation, migration/invasion through downregulation of OIP5-AS1.

**Fig. 8:**
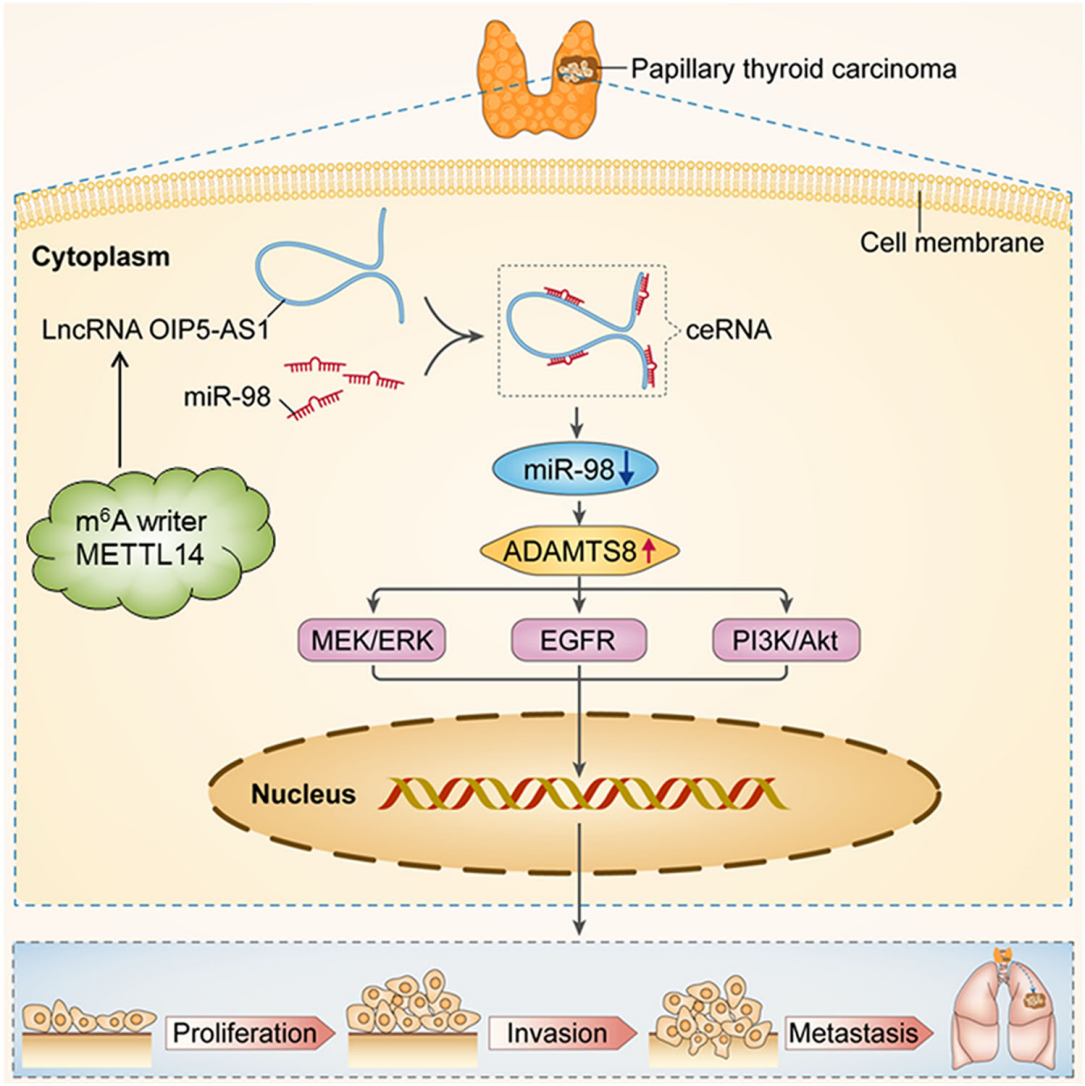
A Schematic diagram of METTL14-mediated lncRNA OIP5-AS1 implicated in the Progression of PTC. METTL14 regulates papillary thyroid carcinoma proliferation and migration/invasion through lncRNA OIP5-AS1-mediated miR-98/ADAMTS8 axis and EGFR, MEK/ERK signaling pathway.

In summary, this study reveals that OIP5-AS1 serves as a sponge for miR-98, which in turn activates ADAMTS8. More importantly, OIP5-AS1 promotes proliferation, migration, and invasion of PTC cells. To our knowledge, this is the first study to show how OIP5-AS1 and ADAMTS8 interact together to promote PTC. Moreover, we also present strong evidence that m^6^A writer METTL14 could bind to OIP5-AS1 and inhibit PTC cell proliferation, migration, and invasion through suppressing OIP5-AS1 expression (Fig. 8). Therefore, we introduce lncRNA OIP5-AS1 and METTL14 as novel candidate markers for PTC diagnosis and therapy.

## Supplementary information

Supplementary Figure legend

Supplementary Figure S1

Supplementary Figure S2

Supplementary Table S1

Supplementary Table S2

## Data Availability

The datasets used and/or analyzed during the current study are available from the corresponding author on reasonable request.

## References

[1] Albores-Saavedra J, Henson DE, Glazer E, Schwartz AM (2007). Changing patterns in the incidence and survival of thyroid cancer with follicular phenotype–papillary, follicular, and anaplastic: a morphological and epidemiological study. Endocr Pathol..

[2] Blomberg M, Feldt-Rasmussen U, Andersen KK, Kjaer SK (2012). Thyroid cancer in Denmark 1943-2008, before and after iodine supplementation. Int J Cancer..

[3] Wang Y, Wang W (2015). Increasing incidence of thyroid cancer in Shanghai, China, 1983–2007. Asia-Pac J Public Health..

[4] Wu DM, Wang S, Wen X, Han XR, Wang YJ, Shen M (2018). LncRNA SNHG15 acts as a ceRNA to regulate YAP1-Hippo signaling pathway by sponging miR-200a-3p in papillary thyroid carcinoma. Cell Death Dis..

[5] Liu J, Eckert MA, Harada BT, Liu SM, Lu Z, Yu K (2018). m(6)A mRNA methylation regulates AKT activity to promote the proliferation and tumorigenicity of endometrial cancer. Nat Cell Biol..

[6] Hou J, Shan H, Zhang Y, Fan Y, Wu B (2020). m(6)A RNA methylation regulators have prognostic value in papillary thyroid carcinoma. Am J Otolaryngol..

[7] Wang X, Feng J, Xue Y, Guan Z, Zhang D, Liu Z (2016). Structural basis of N(6)-adenosine methylation by the METTL3-METTL14 complex. Nature..

[8] Yang X, Zhang S, He C, Xue P, Zhang L, He Z (2020). METTL14 suppresses proliferation and metastasis of colorectal cancer by down-regulating oncogenic long non-coding RNA XIST. Mol Cancer..

[9] Chen X, Xu M, Xu X, Zeng K, Liu X, Pan B (2020). METTL14-mediated N6-methyladenosine modification of SOX4 mRNA inhibits tumor metastasis in colorectal cancer. Mol Cancer..

[10] Wang M, Liu J, Zhao Y, He R, Xu X, Guo X (2020). Upregulation of METTL14 mediates the elevation of PERP mRNA N(6) adenosine methylation promoting the growth and metastasis of pancreatic cancer. Mol Cancer..

[11] Li Z, Jiang P, Li J, Peng M, Zhao X, Zhang X (2018). Tumor-derived exosomal lnc-Sox2ot promotes EMT and stemness by acting as a ceRNA in pancreatic ductal adenocarcinoma. Oncogene..

[12] Gupta RA, Shah N, Wang KC, Kim J, Horlings HM, Wong DJ (2010). Long non-coding RNA HOTAIR reprograms chromatin state to promote cancer metastasis. Nature..

[13] Huarte M, Guttman M, Feldser D, Garber M, Koziol MJ, Kenzelmann-Broz D (2010). A large intergenic noncoding RNA induced by p53 mediates global gene repression in the p53 response. Cell..

[14] Batista Pedro J, Chang Howard Y (2013). Long noncoding RNAs: cellular address codes in development and disease. Cell..

[15] Wang Y, Zhang X, Wang Z, Hu Q, Wu J, Li Y (2018). LncRNA-p23154 promotes the invasion-metastasis potential of oral squamous cell carcinoma by regulating Glut1-mediated glycolysis. Cancer Lett..

[16] Torsin LI, Dragomir MP, Calin GA. Molecular biology of long non-coding RNAs. 2nd ed. Springer; 2019.

[17] Bai Y, Li S (2020). Long noncoding RNA OIP5-AS1 aggravates cell proliferation, migration in gastric cancer by epigenetically silencing NLRP6 expression via binding EZH2. J Cell Biochem..

[18] Dai J, Xu L, Hu X, Han G, Jiang H, Sun H (2018). Long noncoding RNA OIP5-AS1 accelerates CDK14 expression to promote osteosarcoma tumorigenesis via targeting miR-223. Biomed Pharmacother..

[19] Li M, Ning J, Li Z, Fei Q, Zhao C, Ge Y (2019). Long noncoding RNA OIP5-AS1 promotes the progression of oral squamous cell carcinoma via regulating miR-338-3p/NRP1 axis. Biomedicine Pharmacother..

[20] Zhang J, Zhao T, Tian L, Li Y (2019). LncRNA OIP5-AS1 promotes the proliferation of hemangioma vascular endothelial cells via regulating miR-195-5p/NOB1 axis. Front Pharmacol..

[21] Zhang Z, Liu F, Yang F, Liu Y (2018). Kockdown of OIP5-AS1 expression inhibits proliferation, metastasis and EMT progress in hepatoblastoma cells through up-regulating miR-186a-5p and down-regulating ZEB1. Biomedicine Pharmacother..

[22] Deng J, Deng H, Liu C, Liang Y, Wang S (2018). Long non-coding RNA OIP5-AS1 functions as an oncogene in lung adenocarcinoma through targeting miR-448/Bcl-2. Biomedicine Pharmacother..

[23] Mochizuki S, Okada Y (2007). ADAMs in cancer cell proliferation and progression. Cancer Sci..

[24] Porter S, Span PN, Sweep FC, Tjan-Heijnen VC, Pennington CJ, Pedersen TX (2006). ADAMTS8 and ADAMTS15 expression predicts survival in human breast carcinoma. Int J Cancer..

[25] Stokes A, Joutsa J, Ala-Aho R, Pitchers M, Pennington CJ, Martin C (2010). Expression profiles and clinical correlations of degradome components in the tumor microenvironment of head and neck squamous cell carcinoma. Clin Cancer Res..

[26] Li JH, Liu S, Zhou H, Qu LH, Yang JH (2014). starBase v2.0: decoding miRNA-ceRNA, miRNA-ncRNA and protein-RNA interaction networks from large-scale CLIP-Seq data. Nucleic Acids Res..

[27] Agarwal V, Bell GW, Nam JW, Bartel DP (2015). Predicting effective microRNA target sites in mammalian mRNAs. eLife.

[28] Bellucci M, Agostini F, Masin M, Tartaglia GG (2011). Predicting protein associations with long noncoding RNAs. Nat Methods..

[29] Gou Q, Gao L, Nie X, Pu W, Zhu J, Wang Y (2018). Long noncoding RNA AB074169 inhibits cell proliferation via modulation of KHSRP-mediated CDKN1a expression in papillary thyroid carcinoma. Cancer Res..

[30] Wang H, Liang L, Dong Q, Huan L, He J, Li B (2018). Long noncoding RNA miR503HG, a prognostic indicator, inhibits tumor metastasis by regulating the HNRNPA2B1/NF-kappaB pathway in hepatocellular carcinoma. Theranostics..

[31] Wang R, Dong HX, Zeng J, Pan J, Jin XY (2018). LncRNA DGCR5 contributes to CSC-like properties via modulating miR-330-5p/CD44 in NSCLC. J Cell Physiol..

[32] Zhen Q, Gao LN, Wang RF, Chu WW, Zhang YX, Zhao XJ (2018). LncRNA DANCR promotes lung cancer by sequestering miR-216a. Cancer Control..

[33] Shi G, Li H, Gao F, Tan Q (2018). lncRNA H19 predicts poor prognosis in patients with melanoma and regulates cell growth, invasion, migration and epithelial-mesenchymal transition in melanoma cells. Onco Targets Ther..

[34] Li TH, Zhang JJ, Liu SX, Chen Y (2018). Long non-coding RNA taurine-upregulated gene 1 predicts unfavorable prognosis, promotes cells proliferation, and inhibits cells apoptosis in epithelial ovarian cancer. Medicine..

[35] Wang Y, Gu J, Lin X, Yan W, Yang W, Wu G (2018). lncRNA BANCR promotes EMT in PTC via the Raf/MEK/ERK signaling pathway. Oncol. Lett..

[36] Yuan Q, Liu Y, Fan Y, Liu Z, Wang X, Jia M (2018). LncRNA HOTTIP promotes papillary thyroid carcinoma cell proliferation, invasion and migration by regulating miR-637. Int J Biochem Cell Biol..

[37] Zhang XF, Ye Y, Zhao SJ (2018). LncRNA Gas5 acts as a ceRNA to regulate PTEN expression by sponging miR-222-3p in papillary thyroid carcinoma. Oncotarget.

[38] Zhu H, Lv Z, An C, Shi M, Pan W, Zhou L (2016). Onco-lncRNA HOTAIR and its functional genetic variants in papillary thyroid carcinoma. Sci Rep..

[39] Choi GC, Li J, Wang Y, Li L, Zhong L, Ma B (2014). The metalloprotease ADAMTS8 displays antitumor properties through antagonizing EGFR-MEK-ERK signaling and is silenced in carcinomas by CpG methylation. Mol Cancer Res..

[40] Zhang J, Wang P, Dykstra M, Gelebart P, Williams D, Ingham R (2012). Platelet-derived growth factor receptor-alpha promotes lymphatic metastases in papillary thyroid cancer. J Pathol..

