# Supplementary material for: METTL14 promotes tumorigenesis by regulating lncRNA OIP5-AS1/miR-98/ADAMTS8 signaling in papillary thyroid cancer
Source: Cell Death Dis. 2021 Jun 15;12(6):617. doi: 10.1038/s41419-021-03891-6 (PMC8206147; doi:10.1038/s41419-021-03891-6)
Supplement: Supplementary file 1 — Supplementary Figure legend [file 41419_2021_3891_MOESM1_ESM.docx]

**Figure S1.** (**A**) Immunostaining showing that ADAMTS8 expression is higher in PTC tissues than adjacent normal tissues (n=6). Scale bar=200μm. (**B**) The efficiency of OIP5-AS1 konockdown and overexpression were detected by qRT-PCR. Knockdown of OIP-AS1 using two different siRNAs (si-OIP5-AS1-#1, si-OIP5-AS1-#1) or a control siRNA (si-NC) in TPC-1 cells, overexpression of OIP5-AS1 were in K1 cells (n=3). (**C**) The efficiency of ADAMTS8 konockdown and overexpression were detected by qRT-PCR. Knockdown of ADAMTS8 using two different siRNAs (si- ADAMTS8-#1, si- ADAMTS8-#1) or a control siRNA (si-NC) in TPC-1 cells, overexpression of ADAMTS8 were in K1 cells (n=3). (**D**) LncRNA immunoprecipitation assays were performed in lncRNA OIP5-AS1 overexpressing TPC-1 and K1 cells using OIP5-AS1-specific probe and control probe, respectively. The enrichment of OIP5-AS1 and microRNAs was detected by qRT-PCR and normalized to the control probe (n=3). (**E**) Pearson correction of OIP5-AS1 and miR-98 were analyzed (n = 72). (**F**) qRT-PCR analyses the levels of METTL14 in TPC and K1 cells, which were transfected with si-NC/si-OIP5-AS1 and pcDNA3.1/OIP5-AS1 (n=3). ** *p* <0.01 and *** *p* <0.001. n.s=no significance.

**Figure S2.** Immunofluorescence was used to measure ADAMTS8 expression in K1 cells co-transfected with miR-98 inhibitor and si-ADAMTS8, and TPC-1 cells co-transfected with miR-98 mimic and ADAMTS8.
