# Supplementary figures and images for: METTL14 promotes tumorigenesis by regulating lncRNA OIP5-AS1/miR-98/ADAMTS8 signaling in papillary thyroid cancer
Source: Cell Death Dis. 2021 Jun 15;12(6):617. doi: 10.1038/s41419-021-03891-6 (PMC8206147; doi:10.1038/s41419-021-03891-6)

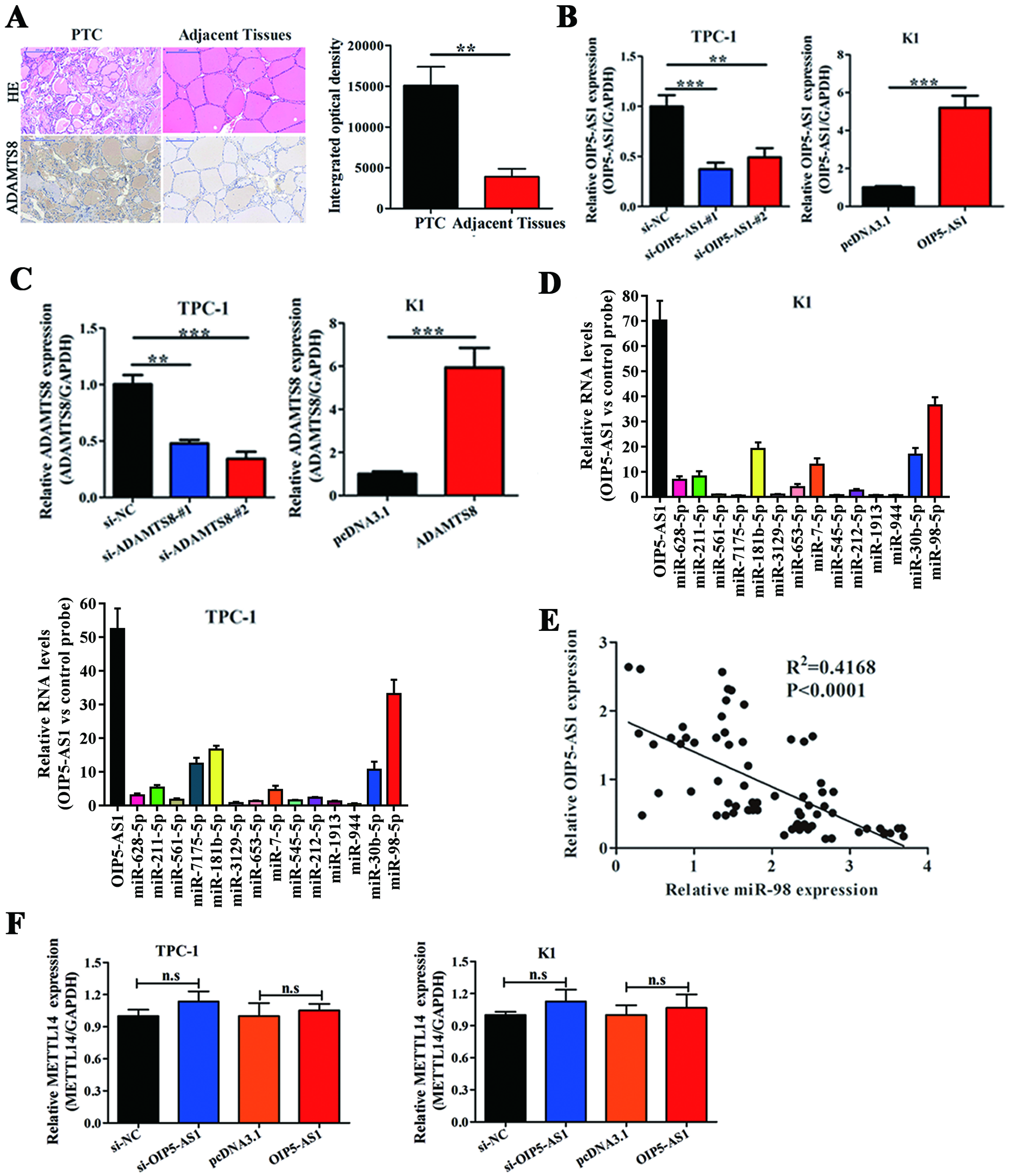

Supplement: Supplementary file 2 — Supplementary Figure S1 [file 41419_2021_3891_MOESM2_ESM.tif]

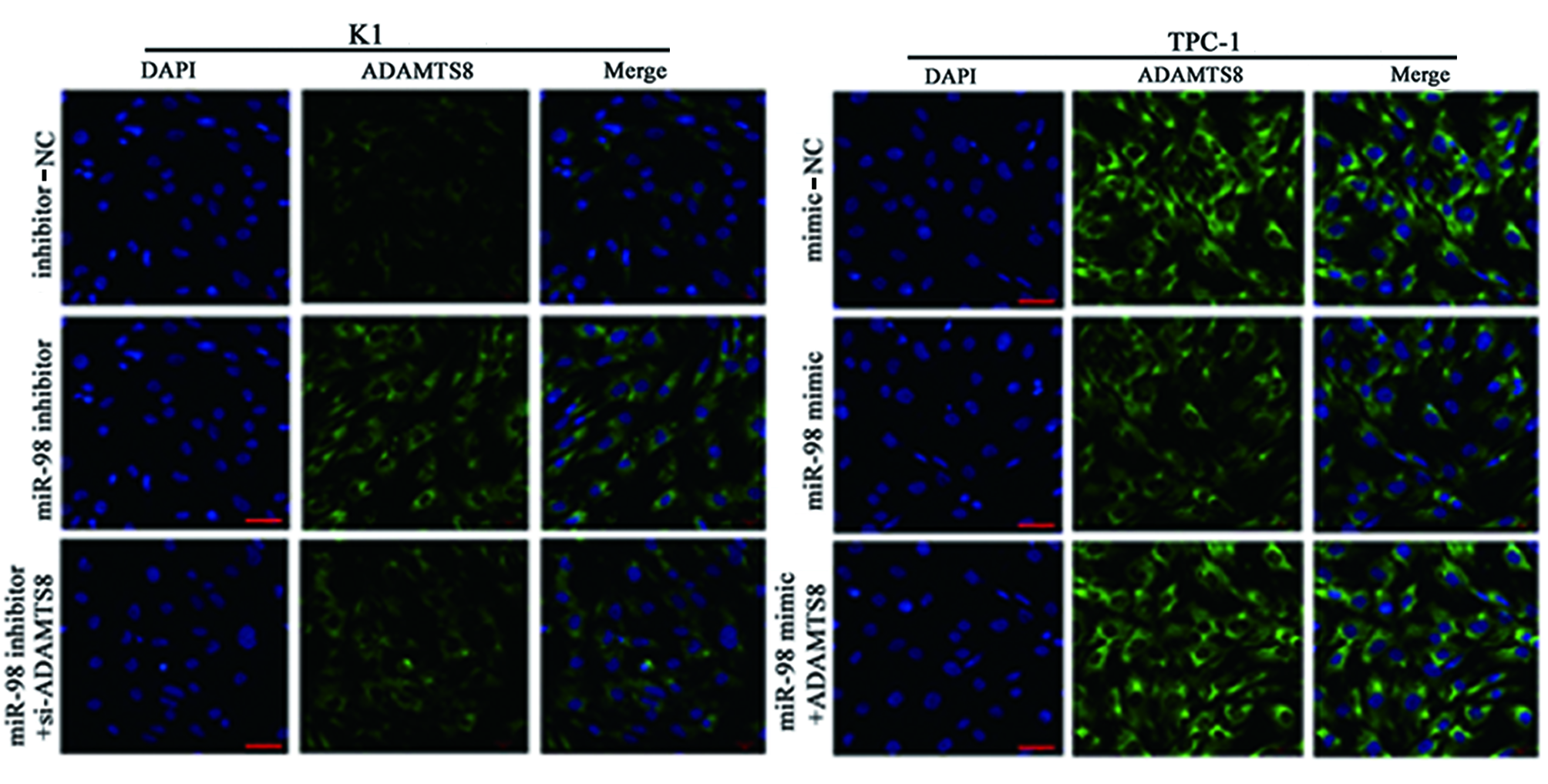

Supplement: Supplementary file 3 — Supplementary Figure S2 [file 41419_2021_3891_MOESM3_ESM.tif]
