# Supplementary material for: METTL14 promotes tumorigenesis by regulating lncRNA OIP5-AS1/miR-98/ADAMTS8 signaling in papillary thyroid cancer
Source: Cell Death Dis. 2021 Jun 15;12(6):617. doi: 10.1038/s41419-021-03891-6 (PMC8206147; doi:10.1038/s41419-021-03891-6)
Supplement: Supplementary file 4 — Supplementary Table S1 [file 41419_2021_3891_MOESM4_ESM.docx]

**Table 1** Clinical characteristics and expressions of LncRNA OIP5-AS1 and ADAMTS8 in 72 papillary thyroid carcinoma patients

|  | LncRNA OIP5-AS1 | | | ADAMTS8 | | |
| --- | --- | --- | --- | --- | --- | --- |
|  | Low  N=30 | High  N=42 | *P* | Low  N=35 | High  N=37 | *P* |
| Age(years) |  |  | 0.953 |  |  | 0.925 |
| ≤45 | 9 | 7 |  | 11 | 5 |  |
| ＞45 | 21 | 35 |  | 24 | 32 |  |
| Gender |  |  | 0.344 |  |  | 0.421 |
| Male | 5 | 8 |  | 6 | 7 |  |
| Female | 25 | 34 |  | 29 | 30 |  |
| Differentiation grade |  |  | 0.225 |  |  | 0.216 |
| Well/moderate | 13 | 18 |  | 15 | 16 |  |
| Poor | 17 | 24 |  | 20 | 21 |  |
| Tumour size |  |  | 0.032^*^ |  |  | 0.043^*^ |
| T1/T2 | 24 | 17 |  | 21 | 20 |  |
| T3/T4 | 6 | 25 |  | 14 | 17 |  |
| Lymph node metastasis |  |  | 0.013^*^ |  |  | 0.028^*^ |
| Negative | 19 | 9 |  | 11 | 17 |  |
| Positive | 11 | 33 |  | 24 | 20 |  |
| TNM stage |  |  | 0.018^*^ |  |  | 0.012^*^ |
| Ⅰ/Ⅱ | 21 | 29 |  | 27 | 23 |  |
| Ⅲ/Ⅳ | 9 | 13 |  | 8 | 14 |  |

Low/high expression was sorted by the sample median value. Pearson χ2 test

**P* < 0.05 was considered to be statistically significant.
