# Supplementary material for: METTL14 promotes tumorigenesis by regulating lncRNA OIP5-AS1/miR-98/ADAMTS8 signaling in papillary thyroid cancer
Source: Cell Death Dis. 2021 Jun 15;12(6):617. doi: 10.1038/s41419-021-03891-6 (PMC8206147; doi:10.1038/s41419-021-03891-6)
Supplement: Supplementary file 5 — Supplementary Table S2 [file 41419_2021_3891_MOESM5_ESM.docx]

| **Table 2. Information on the RT-PCR primer sequence and siRNA sequence** | | |
| --- | --- | --- |
| Name | Primer direction | Sequence |
| OIP5-AS1 | Forward | 5’-TGCGAAGATGGCGGAGTAAG-3’ |
|  | Reverse | 5’- TAGTTCCTCTCCTCTGGCCG -3 |
| ADAMTS8 | Forward | 5’-GTGACCCCAACAAAAGCTGC-3’ |
|  | Reverse | 5’-GGACGTGCCCTAGTTCATGG-3’ |
| METTL14 | Forward | 5’-AGTGCCGACAGCATTGGTG-3’ |
|  | Reverse | 5’-GGAGCAGAGGTATCATAGGAAGC-3’ |
| GAPDH | Forward | 5’-TGTGAACGGATTTGGCCGTA-3’ |
|  | Reverse | 5’-GGTCTCGCTCCTGGAAGATG-3’ |
| miR-98 | Forward | 5’-GATTCTGCTCATGCCAGGGT-3’ |
|  | Reverse | 5’-GCCACACACCAGGGAAAGTAG-3’ |
| U6 | Forward | 5’-CGCTTCGGCAGCACATATAC-3’ |
|  | Reverse | 5’-AAATATGGAACGCTTCACGA-3’ |
| si-METTL14-1 |  | 5’-GCATTGGTGCCGTGTTAAA-3’ |
| si-METTL14-2 |  | 5’-GGATGAACTAGAAATGCAA-3’ |
| si-OIP5-AS1-1 |  | 5’- GGACUUGUCUGAUUAGGUUTT -3’ |
| si-OIP5-AS1-2 |  | 5’- CCUUUGGACUUACAGGAAUTT -3’ |
| si-ADAMTS8-1 |  | 5’- GCAGACATCGGGACCATTT-3’ |
| si-ADAMTS8-1 |  | 5’-GCAGCAGTGTGAGAAGTAT-3’ |
